# Study on the Regulatory Mechanisms of Carapace Marking Formation in *Marsupenaeus japonicus*

**DOI:** 10.3390/ani15050727

**Published:** 2025-03-04

**Authors:** Panpan Wang, Jiawei Zhu, Huanyu Chen, Qingyuan Hu, Zhenxiang Chen, Wenjia Li, Ting Yang, Jin Zhu, Binlun Yan, Huan Gao, Chaofan Xing

**Affiliations:** 1 Jiangsu Key Laboratory of Marine Biotechnology, Jiangsu Ocean University, Lianyungang 222005, China; wangpp90@jou.edu.cn (P.W.); 14761056889@163.com (J.Z.); mesace0719@163.com (H.C.); 18107246837@163.com (Q.H.); 18860810093@163.com (Z.C.); lwj6668882023@163.com (W.L.); yt12341234yt@163.com (T.Y.); 13151705111@163.com (J.Z.); yanbinlun1962@163.com (B.Y.); huanmr@163.com (H.G.); 2 Co-Innovation Center of Jiangsu Marine Bio-Industry Technology, Jiangsu Ocean University, Lianyungang 222005, China; 3 Jiangsu Institute of Marine Resources Development, Jiangsu Ocean University, Lianyungang 222005, China; 4 The Jiangsu Provincial Infrastructure for Conservation and Utilization of Agricultural Germplasm, Nanjing 210014, China

**Keywords:** *Marsupenaeus japonicus*, markings, microRNA, crustacyanin, regulatory mechanism

## Abstract

There are two phenotypes in the natural populations of *Marsupenaeus japonicus*, which is an ideal model for studying the formation of markings and body color in crustaceans. In a previous study, we used comparative transcriptome technology to screen some functional genes related to body color regulation. Here, high-throughput sequencing technology was used to perform microRNA (miRNA) sequencing analysis on the exoskeleton of *M. japonicus* with two types of carapace markings, and functional studies of related genes were performed. A total of 687 mature miRNAs belonging to 135 miRNA families were identified in this study, and 111 novel miRNAs were found. Through stringent screening conditions, a total of 18 differentially expressed miRNAs were identified, including 14 with upregulated expression and 4 with downregulated expression. Multiple target genes were predicted for almost all of the differentially expressed miRNAs. The expression levels of several target genes, such as those related to cytoplasmic microtubule organization, transmembrane transportation, and signal transduction, were confirmed using qRT-PCR. This study revealed that both the *CRCN A2* and *CRCN C1* genes were highly expressed in type I individuals, while the expression levels of their related miRNAs in type I individuals were lower than those in type II individuals, which is consistent with the mechanism of miRNAs negatively regulating mRNA expression. Through interference with the *CRCN A2* and *CRCN C1* genes, a clear regulatory relationship was found between the two genes, and the dendritic xanthophores in the carapace of *M. japonicus* gradually changed from bright yellow to dark black, with obvious shrinkage. In summary, our studies provide references for the regulatory mechanisms of marking formation in *M. japonicus*.

## Simple Summary

In this study, the two types of *Marsupenaeus japonicus* were taken as the research objects, and the functional genes related to marking formation were screened by high-throughput sequencing technology. This study identified 18 differentially expressed miRNAs, and some of their functions were annotated as epidermal structure, transmembrane transport, and intermediate fibers. The expression characteristics of *CRCN A2* and *CRCN C1* genes were analyzed by fluorescence quantitative PCR, and RNA interference experiments were carried out to clarify that they are involved in regulating the distribution of pigment cells in the carapace of *M. japonicus*. This study provides important data support for revealing the molecular regulation mechanism of markings and body color formation in crustaceans.

## 1. Introduction

*Marsupenaeus japonicus*, also known as nine-jointed shrimp, is an economically farmed prawn worldwide [1]. The present study found that *M. japonicus* has two phenotypes in natural populations, mainly reflected in carapace markings, of which type I has longer markings [2,3,4,5]. The markings of animals are the result of different locations of pigmentation. The type and quantity of animal chromatophores, the chromatophore size, and the strength of guanine crystals in iridocytes all affect the body color and vividness of animals [6,7]. The phenotypic characteristics of an animal are essential for its survival, reproduction, and information exchange [8,9]. The two types of markings in the natural population of *M. japonicus* are ideal materials for studying the formation of crustaceans’ markings.

MicroRNAs are endogenous small noncoding RNA molecules of approximately 20–24 nucleotides in length that play key roles in various biological processes by promoting mRNA degradation or inhibiting mRNA translation [10,11,12]. Studies have shown that miRNA–mRNA interactions are related to several important physiological processes in organisms, including cell apoptosis, cell proliferation, embryonic development, and epidermal pigmentation [13,14,15]. Many studies have shown that miRNAs play important regulatory roles in body color formation or epidermal pigmentation in aquatic animals [11,14]. Dong et al. used small RNA technology to sequence and analyze three different epidermal tissues of carp, identified 164 differentially expressed miRNAs, and predicted multiple target genes associated with pigmentation; small RNA analysis of two color variants revealed 32 differentially expressed miRNAs [16,17]. miR-196d was found to potentially regulate melanocyte synthesis and movement indirectly by targeting the *myh7* (myosin-7) gene [18,19]. This study revealed that miRNA-196a and miRNA-206 are involved in the carp epidermal pigmentation process by regulating the target genes *mifta* and *Mc1r*, respectively. Wu et al. reported that miR-495 affects rainbow trout skin color by regulating the expression of the mc1r gene [20].

Studies have shown that crustacean erythrophores contain high levels of carotenoids [21,22,23]. Previous studies have revealed that the carapace markings of *M. japonicus* were dominated by erythrophores and that the astaxanthin level (95.8%) in the carapace was significantly higher than those of other pigments [5]. Carotenoid-binding proteins in aquatic crustaceans stabilize carotenoids, especially astaxanthin [24,25,26]. Crustacyanin is a unique carotenoid-binding protein in crustaceans. It is polymerized by eight dimers of β-crustacyanin (composed of CRCN-A and CRCN-C subunits) and can covalently bind to ingested astaxanthin to form a complex, which leads to a change in the conformation of astaxanthin, thus affecting the color of the crustacean exoskeleton [22,27,28,29,30]. Crustacyanin was first isolated from the hard shell of the European lobster (*Homarus gammarus*) [31]. To date, crustacyanin subunits have been obtained from more than 10 species of prawn, including *Penaeus monodon*, *Macrobrachium rosenbergii*, and *Penaeus merguiensis*, and all of these subunits are involved in the development of body color [28,29,32,33,34,35]. Our team previously cloned the four subunits of the *CRCN* gene A1, A2, C1, and C2 from *Palaemon carinicauda*. After RNA interference, the blue pigment on the carapace, trunk, and tail fan of the individuals in the experimental group essentially disappeared [33]. The astaxanthin molecules were released, causing the prawn to turn red. Dietary carotenoids exist in the form of mixed micelles and are absorbed by intestinal cells through the membrane bilayer [36,37]. The transmembrane transport process requires the mediation of apolipoproteins, scavenger receptors, and other transporters. The lipophilicity of carotenoids determines that the transport process requires the participation of lipoproteins. Once apolipoproteins are loaded with carotenoids, they can be targeted for transport to different tissues [38]. Lin et al. screened the skin transcriptomes of red, yellow, and transparent strains of *Neocaridina denticulata* and identified several differentially expressed genes associated with pigment granule transport and precipitation, such as apolipoprotein D and carotenoid-binding protein [39].

In our previous study, we used transcriptome technology to identify some functional genes associated with markings, including crustacyanin and apolipoprotein D. The levels and amino acid structure composition of crustacyanin and *ApoD* were different between two types of *M. japonicus* [5]. In this study, high-throughput sequencing technology was used to identify miRNAs associated with the formation of markings in the exoskeleton of two types of prawns, providing important data for revealing the molecular regulatory mechanisms involved in the formation of markings and body color in crustaceans.

## 2. Materials and Methods

### 2.1. Sample Collection

The *M. japonicus* used in the experiments was from the experimental pond located in Ganyu County, Jiangsu Province, China (34°58′17.30″ N, 119°11′53.70″ E). The average body weight of the type I *M. japonicus* was 7.42 ± 2.45 g, and the average body weight of the type II *M. japonicus* was 6.32 ± 2.25 g. All the prawns were reared for one week in a controlled environment at 25 ± 1 °C and a salinity value of 28‰. During the temporary rearing period, the air was continuously aerated, and the prawns were fed twice daily with commercial pellets (Fuxing, Shanghai, China), which contained 39% protein, 6% crude fiber, and 11% moisture. The feeding amount was 5% of the body weight, and the feed residues and manure were removed 2 h after each feeding. After the end of the temporary rearing, the experimental prawns were euthanized using an anesthetic. The exoskeleton tissues of the carapace were collected from 40 *M. japonicus* of each phenotype, and the intestines, gills, muscle, exoskeleton, stomach, ventral nerve cord, eyestalk, and hepatopancreas tissues were collected from another six individuals. The tissues were immediately snap-frozen in liquid nitrogen for total RNA extraction.

### 2.2. RNA Extraction and cDNA Synthesis

The carapace exoskeleton samples from the 40 type I and 40 type II *M. japonicus* were divided into eight groups, and total RNA was extracted from eight groups: three RNA samples were used for miRNA sequencing analysis, and the remaining samples were used for conventional PCR and fluorescence quantitative PCR. The RNA was extracted from the samples using TRIzol reagent (Takara, Dalian, China). RNA purity and integrity were checked using the NanoPhotometer^®^ spectrophotometer (IMPLEN, Westlake Village, CA, USA) and the Agilent 2100 Bioanalyzer (Agilent Technologies, Santa Clara, CA, USA), respectively.

### 2.3. Construction and Sequencing of the miRNA Library

After the samples were subjected to analysis, equal amounts (concentration × volume) of RNA from different individuals in each group were mixed, and the NEBNext^®^Ultra^TM^ small RNA sample prep kit (NEB, New England Biolab, Ipswich, MA, USA) for Illumina^®^ was used for small RNA library construction. Specifically, by using special structures at the 3′ and 5′ ends of small RNA, linkers were added to both ends of the small RNA, and cDNA was subsequently synthesized via reverse transcription. After PCR amplification, the target DNA fragments were separated via polyacrylamide gel electrophoresis (PAGE), and gel extraction was used to obtain the small RNA library. After the construction of the library, a Qubit 2.0 instrument (Life Technologies, Carlsbad, CA, USA) was used for preliminary quantification. The library was diluted to 1 ng/µL. The insert size of the library was subsequently determined using a high-sensitivity Agilent 2100 Bioanalyzer (Agilent Technologies, Santa Clara, CA, USA), and the effective concentration of the library was quantified via qPCR. After passing the library check, sequence clusters were generated by the cBot Cluster Generation System using TruSeq PE Cluster Kit v4-cBot-HS (Illumia, San Diego, CA, USA) and then sequenced on the Illumina Hiseq X-ten (Illumia, San Diego, CA, USA) high-throughput sequencing platform.

### 2.4. miRNA Bioinformatics Analysis

To ensure the accuracy of subsequent data analysis, quality control was performed on the original data to remove reads with low-quality values; reads with unrecognized base N contents of 10% or greater, without 3′ adapter sequences, with 5′ adapter contamination, containing polyA, or shorter than 18 nucleotides were removed. Bowtie2 (v.2.4.1) was used for sequence alignment of the clean reads with the Silva, GtRNAdb, Rfam, and Repbase databases, and the ribosomal RNA (rRNA), transfer RNA (tRNA), small nuclear RNA (snRNA), small nucleolar RNA (snoRNA), and repetitive sequences were filtered to obtain unannotated reads containing miRNAs. First, the unannotated reads were aligned with the transcriptome of *M. japonicus* using Bowtie2 (v.2.4.1), and the clean reads from the alignment were used for subsequent analysis. To obtain the potential miRNA, the predicted miRNA hairpins were compared against miRNA precursor sequences from miRBase22.0 [40]. The miREvo and miRDeep2 were integrated to predict novel miRNA [41,42].

### 2.5. Differentially Expressed miRNA Analysis and miRNA Target Prediction

miRNA expression levels were estimated by TPM (transcript per million) referenced to Zhou’s criteria [43]. Differential expression analysis was performed using the DESeq2 package [44]. Those miRNAs with padj < 0.05 and a fold change ≥ 2 were identified as DEMs. The intersection of target genes predicted by miRanda and RNAhybrid was considered to be the final target gene [45,46]. According to the correspondence between miRNAs and their target genes, the target gene sets of DEMs were characterized by Gene Ontology enrichment analysis using the Goseq method, and the q value was set to <0.05 to ensure the reliability of the analysis results [47]. The top 20 enrichment results were visualized using the OmicShare tool (https://www.omicshare.com/tools, accessed on 1 December 2024).

### 2.6. Validation of miRNA Expression by Real-Time Quantitative PCR

Using U6 as an internal reference gene, the relative expression of miRNA was detected by fluorescence quantitative PCR. There are five biological replicates in each group of samples and three technical replicates in each sample. Primers were designed using Primer Premier 5.0 based on mature miRNA sequence information obtained from Small RNA sequencing (Table S1). The relative expression of the target gene was calculated by the 2^−ΔΔCt^ method. SPSS 26.0 software (SPSS, Inc., Chicago, IL, USA) was used for one-way ANOVA, and Duncans’ multiple comparison was used to test the significance of the mean value.

### 2.7. Tissue Expression Characteristics of Crustacyanin Genes

RNA was extracted from the epidermis, intestine, gill, muscle, stomach, abdominal nerve cord, eye stalk, and hepatopancreas of *M. japonicus*, and single-stranded cDNA was synthesized by PrimeScriptTM first-strand cDNA synthesis kit (Takara, Dalian, China). The expression levels of *CRCN A2* and *CRCN C1* genes were analyzed by qRT-PCR and 2^−ΔΔCt^ methods. 18S rRNA was used as an internal reference gene.

### 2.8. RNAi Experiment of Crustacyanin Genes

RNAi double-stranded DNA template was synthesized using a conventional PCR DNA template. Then purify dsRNA according to the instructions of the T7 RNAi Transcription Kit (Vazyme, Nanjing, China). In order to carry out the RNAi experiment, 120 healthy *M. japonicus* were divided into the *CRCN A2* group, *CRCN C1* group, and control group, with 40 shrimps in each group. The 10 μL of corresponding interfering reagent or normal saline was injected into the pericardial cavity of each shrimp. Then, the epidermal tissues of nine shrimps from each group were collected at 0 d, 2 d, 4 d, and 7 d after injection, and all samples were stored in Trizol for total RNA extraction. The expression changes of related genes were detected by qRT-PCR. The effect of crustacyanin silencing on the pigment cells of *M. japonicus* was observed by a Leica DM500 microscope (Leica Microsystems GmbH, Wetzlar, Germany).

## 3. Results

### 3.1. Analysis and Identification of Small RNAs

To identify differentially expressed miRNAs, two miRNA libraries of epidermal tissues were constructed and sequenced. All sequencing data are shown in Table 1. We obtained 70,827,435 original reads from two small RNA libraries. The raw reads were filtered to remove 5’ and 3′ joints, no load, and poly (A) reads. Finally, 32,639,415 clean reads were obtained from the type I library and 36,456,852 clean reads were obtained from the type II library. The average percentage of Q20 and Q30 bases in each sample was 99.59% and 98.5%, respectively.

**Figure 1:**
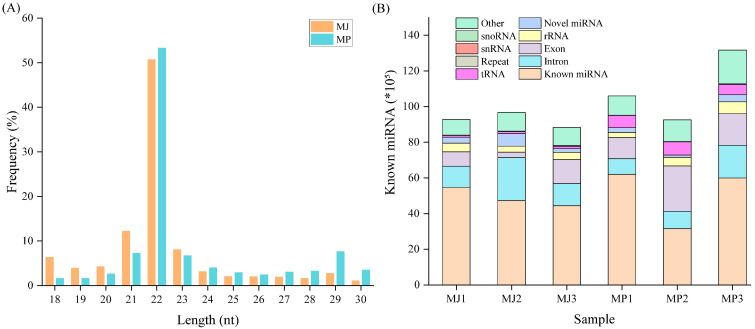
Distribution of miRNA lengths (**A**) and the ncRNAs identified in the different samples (**B**).

The clean reads of each sample were screened, and sRNAs within a certain length range were selected for subsequent analysis. Most of the mature miRNAs were 21~23 nt in length, with the greatest number of small RNAs being 22 nt. Based on the existing database, different types of ncRNAs were identified, of which known miRNAs accounted for more than half (Figure 1). A total of 687 mature miRNAs and 1636 miRNA precursors were identified from all the sRNA libraries. Moreover, on the basis of the archetypal hairpin structure of the miRNA precursors, 111 new miRNAs with hairpin structures were predicted.

### 3.2. Differentially Expressed miRNA Analysis

**Figure 2:**
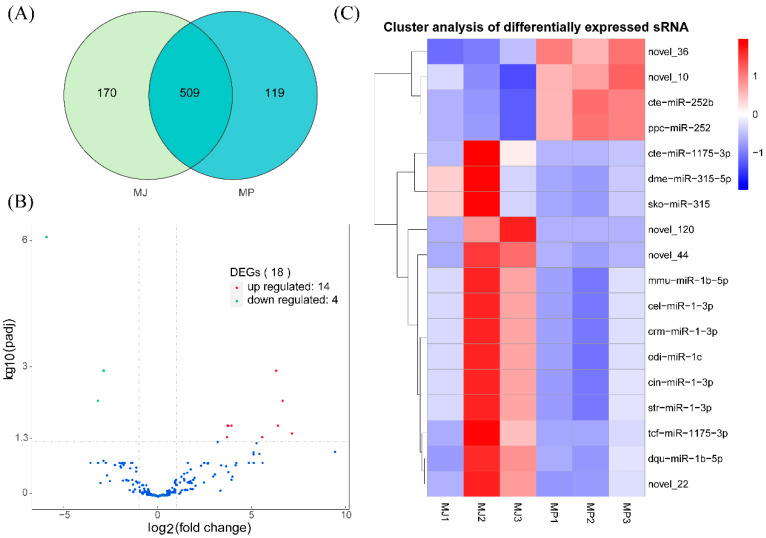
Distribution characteristics of miRNA expression in two libraries of epidermal tissues, (**A**) miRNA numbers of two groups, (**B**) MA plot of differential miRNA expression levels between two group samples, and blue, red, and green dots represent non-significant, up-regulated, and down-regulated miRNAs, respectively. (**C**) A heatmap of all DEMs shows the expression levels, which ranged from low to high, from blue to red.

The expression levels of known miRNAs and novel miRNAs in each sample were determined and normalized by transcripts per million (TPM). Unique and consensus analyses were performed on the miRNAs from the two groups. The result showed that the two groups shared 509 miRNAs, the type I group contained 170 miRNAs, and the type II contained 119 miRNAs (Figure 2A). The differentially expressed genes were screened based on the miRNA expression levels of the samples obtained from each group, and a total of 18 differentially expressed miRNAs were identified, including 14 up-regulated and 4 down-regulated miRNAs (Figure 2B). Some miRNAs had similar *p* values (such as cte-miR-252b, ppc-miR-252, crm-miR-1-3p, dme-miR-315-5p, etc.); therefore, they overlapped in the plots. Five of the differentially expressed miRNAs were novel miRNAs, of which three miRNAs were upregulated (novel 22, novel 44, and novel 120), and two miRNAs were downregulated (novel 10 and novel 36) (Figure 2C).

### 3.3. miRNA Target Gene Prediction and Functional Analysis

miRanda v3.3a and RNAhybrid v2.2 software was used to predict target genes of the differentially expressed miRNAs to explore their potential regulatory functions. A total of 483 target genes were predicted from the 18 differentially expressed miRNAs (Table S1). All the differentially expressed miRNAs were predicted to have multiple target genes, such as crm-miR-1-3p, mmu-miR-1b-5p, novel 120, sko-miR-315, and odi-miR-1c identified separately 72, 61, 35, 31, and 28 target genes. The tcf-miR-1175-3p has target genes related to cytochrome synthesis, and novel-44 and sko-miR-315 both have target genes related to the formation of the exoskeleton structure (Table S1).

**Figure 3:**
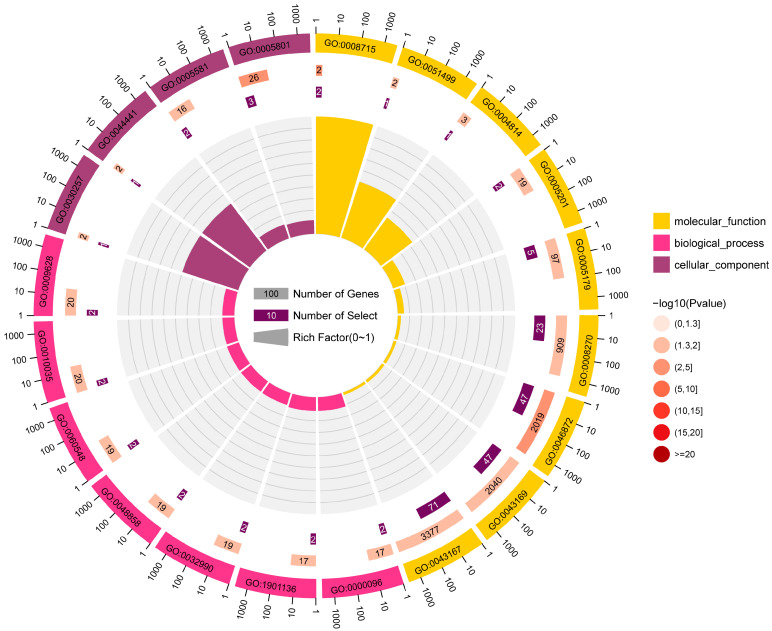
GO enrichment analysis of predicted target genes for differentially expressed miRNAs.

Gene Ontology enrichment analysis was performed on the target genes of the differentially expressed miRNAs to study the distribution of the candidate target genes. The target genes were enriched in eight cellular components, 27 biological processes, and 203 molecular functions. The majority of the biological process entries were related to response to abiotic stimulus (GO:0009628), response to inorganic substance (GO:0010035), and cell part morphogenesis (GO:0032990). The cis-Golgi network (GO:0005801), collagen trimer (GO:0005581), and ciliary part (GO:0044441) were the most abundant terms in the cellular component. In the molecular function category, most terms included ion binding (GO:0043167), cation binding (GO:0043169), and metal ion binding (GO:0046872) (Figure 3).

### 3.4. Verification of Differential miRNA by qRT-PCR

**Figure 4:**
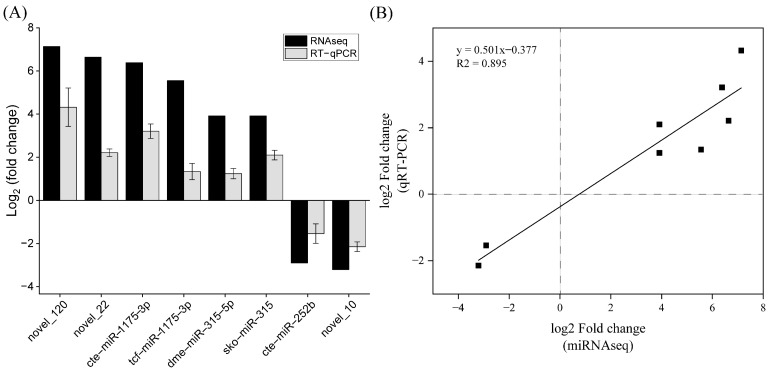
Expression of miRNAs quantified with qRT-PCR. Comparison of fold change in expression between qRT-PCR and RNA-seq data (**A**). Regression analysis between qPCR and miRNA-seq data (**B**).

To verify the reliability and accuracy of differentially expressed miRNA obtained by high-throughput sequencing and bioinformatics analysis, eight differentially expressed miRNA including aae-miR-281-5p, hme-miR-2788-3p, cin-miR-7-5p, mse-miR-281, bmo-miR-281-5p, cte-miR-281, tca-miR-2788-3p, and novel-miR-41 were selected from the sequencing results and analyzed by qRT-PCR. The results showed (Figure 4) that the results of qRT-PCR analysis were consistent with those of miRNA-seq in the changing trend of expression quantity, and the correlation coefficient of regression analysis of the two methods was 0.895, which indicated the reliability of high-throughput miRNA sequencing.

### 3.5. The Expression Characteristics of Crustacyanin Genes

**Figure 5:**
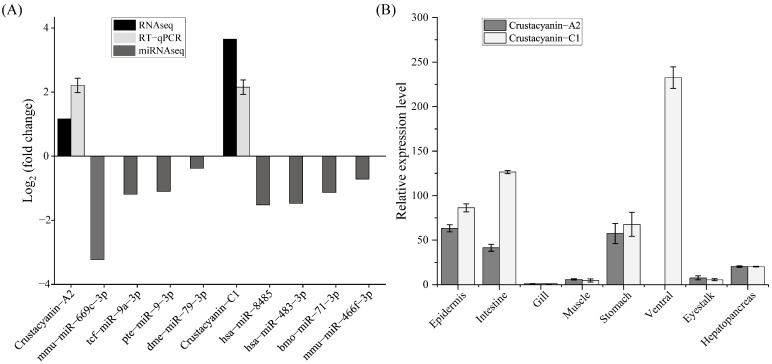
Expression characteristics of the crustacyanin gene. mRNA and miRNA expression levels of epidermal tissues (**A**) and tissue expression levels (**B**) of *CRCN A2* and *CRCN C1*.

This study found that *CRCN A2* and *CRCN C1* genes were highly expressed in type I individuals (Figure 5A). Four miRNAs were identified whose target genes were related to *CRCN A2*, including mmu-miR-669c-3p, tcf-miR-9a-3p, pte-miR-9-3p, and dme-miR-79-3p. Four miRNAs associated with *CRCN C1* were hsa-miR-8485, hsa-miR-483-3p, bmo-miR-71-3p, and mmu-miR-466f-3p. The quantitative PCR results revealed that the expression levels of the above eight miRNAs in type I individuals were lower than those in type II individuals, which was also in agreement with the finding that miRNAs negatively regulate mRNA expression. Tissue expression analysis revealed that the expression level of both crustacyanin genes was the highest in the ventral nerve cord, followed by the intestinal tract, exoskeleton, and gastric tissues, and the expression level in the gills was the lowest (Figure 5B).

### 3.6. Effects of Crustacyanin Gene Silencing on the Expression of Related Genes and Chromatophores of the Carapace

**Figure 6:**
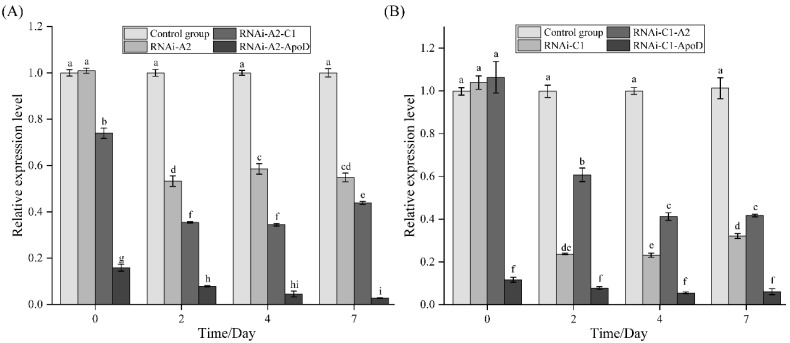
Effects of RNA interference on gene expression in epidermal tissues. (**A**) Effects of *CRCN A2* RNA interference on *CRCN C1* and *ApoD* gene expression. (**B**) Effects of *CRCN C1* RNA interference on *CRCN A2* and *ApoD* gene expression. A significant difference between groups at *p* < 0.05 is indicated by different words.

After siRNA was injected into *M. japonicus,* the expression levels of the *CRCN A2* and *CRCN C1* genes in the interference group and the control group were determined using qRT–PCR after 2 d, 4 d, and 7 d of interference. The results revealed that 48 h after *CRCN A2* siRNA injection, the expression level of the *CRCN A2* gene in the intervention group had decreased by 53% and was significantly lower than that in the control group, and the expression level was still low after 7 d of interference. In addition, the expression levels of the *CRCN C1* and *ApoD* genes were both significantly decreased (Figure 6A). The results revealed that 48 h after *CRCN C1* siRNA injection, the expression level of the *CRCN C1* gene in the intervention group had decreased by 77%, and the expression levels of the *CRCN A2* and *ApoD* genes were both significantly decreased (Figure 6B).

**Figure 7:**
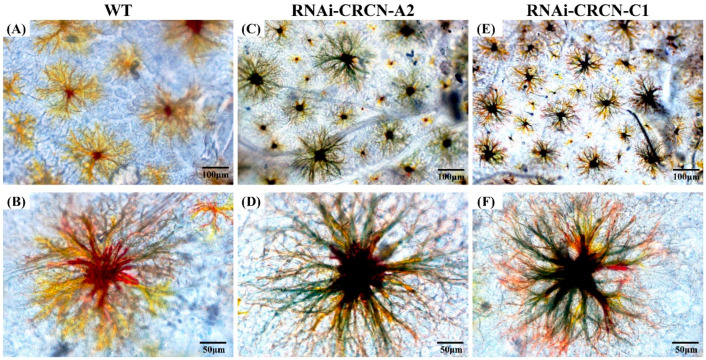
Effects of *CRCN A2* and *CRCN C1* RNA interference on the characteristics of chromatophores in the carapace. (**A**,**B**) represent the control group, (**C**,**D**) represent the effect of *CRCN A2* RNA interference on the chromatophores, and (**E**,**F**) represent the effect of *CRCN C1* RNA interference on the chromatophores.

After interfering with the *CRCN A2* and *CRCN C1* genes, the characteristic changes in the chromatophores of the carapace were observed under a light microscope (Figure 7). In terms of the markings on the carapace of *M. japonicus* in the control group, the xanthophores exhibited a dendritic morphology, and the overall color was bright yellow (Figure 7A,B). After *CRCN A2* interference, the xanthophores in the carapace essentially lost their original color and appeared dark black as a whole, while the cells shrunk to a certain extent (Figure 7C,D). After *CRCN C1* interference, the xanthophores further shrunk, and the overall dark black color became more evident (Figure 7E,F).

## 4. Discussion

Animal body color is a rich and diverse phenotypic trait that has many adaptive functions, such as camouflage, photoprotection, species identification, concealment to confuse predators, and the release of courtship signals [31,48]. Animal markings and body color are mainly the result of the reflection, scattering, absorption, and diffraction of light by chromatophores (melanocytes, xanthophores, iridocytes, etc.) in the skin [49,50]. The distribution characteristics of various pigments in the animal exoskeleton are often affected by nutritional, physiological, environmental, and genetic conditions. Genetics is the most fundamental factor affecting skin pigmentation, and a variety of genes play important regulatory roles in the development of body color [51,52]. In our previous study, we conducted a comparative transcriptomic analysis of the exoskeleton tissues of the carapaces of the two types of *M. japonicus* and preliminarily identified some genes that may be involved in the regulation of body color [5]. To further elucidate the molecular mechanisms underlying the formation of the carapace markings of *M. japonicus*, miRNA technology was used in the present study to compare and analyze the carapaces of the two types of *M. japonicus*, and functional studies were conducted on the different isoforms of the crustacyanin gene. We hope to lay a theoretical foundation for future in-depth investigations of the regulatory mechanisms of crustacean body color.

miRNAs are important posttranscriptional regulatory factors. The different degrees of complementary base pairing with target genes can lead to different regulatory mechanisms of miRNAs on target genes. miRNAs are completely complementary to the bases of the target genes and can guide the splicing of target gene mRNAs. When the bases are not completely complementary or the degree of complementarity is low, miRNA has an inhibitory effect on the translation of target gene mRNAs [12,53]. Chiang et al. reported that animal miRNAs and their target gene mRNAs do not completely match the bases in the 3′-UTR, thus regulating the expression of various functional genes through the translational inhibition of target gene mRNAs [54,55]. There is a growing body of evidence indicating that miRNA dysregulation affects the differentiation and pigmentation of the animal exoskeleton [19,56,57]. Most of the mature miRNAs obtained in this study were 21~23 nt in length, with the greatest number of small RNAs containing 22 nt. This finding is similar to the results of most miRNA studies in marine animals. This length is the typical length of pre-miRNA after shearing by RNase III Dicer [58,59,60,61]. Some identified miRNAs, including miR-1175-3p, miR-750, miR-993, etc., have also been found to be highly expressed in other species, such as *Fenneropenaeus penicillatus* [61], *Litopenaeus vannamei* [62,63], *Eriocheir sinensis* [64], *P. monodon* [65], and *M. rosenbergii* [66].

Through target gene prediction, we further explored the potential relationships between miRNAs and genes. In this study, a total of 18 differentially expressed miRNAs were identified and 483 target genes were predicted, with multiple target genes predicted for each differentially expressed miRNA, suggesting a complex gene expression regulatory network between miRNAs and mRNAs. This study revealed significantly upregulated expression of novel-miR-44, which was functionally annotated as a structural component of the exoskeleton. Among the physiological influences on the cuticle of crustaceans, the molting cycle can change the composition of the cuticle [67]. The product of the *Drosophila* yellow gene, a secreted protein, is a structural component of the pigmented cuticle [68]. The above studies suggest that there is a close relationship between exoskeleton-related genes and pigmentation. The overall marking patterns of the two types of *M. japonicus* were quite similar. The differences between the two were mainly in the completeness of the carapace markings. It was speculated that the completeness of the markings of *M. japonicus* was also related to the transfer of pigment granules. Dietary carotenoids exist in the form of mixed micelles and are absorbed by intestinal cells through the membrane bilayer. The transmembrane transport process requires mediation by apolipoproteins, scavenger receptors, and other transporters [36]. Huang et al. analyzed solute carrier protein family genes from the carapace of *Neocaridina denticulata sinensis* with three pigmentation phenotypes (red, yellow, and chocolate) and reported that in chocolate-colored individuals, the expression levels of ABC transporters (*abcg1*, *abcg14*) and sarcoplasmic calcium-binding proteins were significantly higher than those in the other individuals [37]. miR-1175-3p was identified in this study, which indicates that its target base has a transmembrane transport function and may be involved in the transport of pigment granules. After pigment synthesis, chromatophores affect the body color of animals by aggregating or dispersing pigment granules under the action of cytoskeleton microtubules and microfilaments [69,70]. The expression of both novel-120 and miR-1c identified in this study was upregulated. The two were annotated as cytoplasmic microtubule tissues and intermediate fibers, respectively, and may be involved in the aggregation or dispersion of pigment granules, thus affecting prawn body color.

Chitocyanin is a unique carotenoid-binding protein in crustaceans. There are five precursor subtypes, A1, A2, A3, C1, and C2, all of which belong to the lipocalin family [32]. In the previous study, we found that crustacyanin genes were differential expressions between the two types of *M. japonicus*, and the amino acid sequences were different, indicating the two genes played an important role in the stripe formation of *M. japonicus* [5]. Tissue expression analysis of the *CRCN A2* and *CRCN C1* genes revealed that the expression of both the *CRCN A2* and *CRCN C1* genes was highest in the ventral nerve cord, which was similar to the results in *P. carinicauda* and *Macrobrachium nipponense* [33,71]. In this study, four miRNAs whose target genes were related to *CRCN A2* and *CRCN C1* were annotated, and the expression levels of the miRNAs and mRNAs were significantly negatively correlated, which is consistent with the mechanism of miRNAs negatively regulating mRNA expression. Through interference treatment of the *CRCN A2* and *CRCN C1* genes, we found a clear regulatory relationship between the two genes, which also exerted certain inhibitory effects on the expression of the apolipoprotein gene. As a main component of lipoproteins, once apolipoproteins are loaded with carotenoids, they can be targeted for transport to different tissues [38]. Studies have shown that there is a potential functional relationship between crustacyanin and apolipoproteins, which are jointly involved in the formation of markings or body color. Functional studies of the crustacyanin gene also confirmed this inference. In this study, after *CRCN A2* or *CRCN C1* interference, the number of xanthophores in the carapace of *M. japonicus* gradually changed from bright yellow to dark black, and obvious shrinkage occurred. Several studies have reported similar phenomena in *P. monodon*, *M. rosenbergii*, and *P. carinicauda* [32,33,34].

## 5. Conclusions

In this study, we performed microRNA analysis on the exoskeleton of *M. japonicus*, and identified several differentially expressed miRNAs, some of which were annotated as epidermal structure, transmembrane transport, intermediate fibers, and so on. At the same time, combined with the results of comparative transcriptome between two types of *M. japonicus*, we further studied the expression characteristics and functions of *CRCN A2* and *CRCN C1* genes, and found that these genes were involved in regulating the distribution of chromatophores in the carapace. The structural and functional differences between two crustacyanin genes still need to be further explored. In addition, it is still unknown whether the phenotypic differences between the two types of *M. japonicus* affect their mating, reproduction and information exchange. In conclusion, our study provides important data for revealing the molecular regulatory mechanisms involved in markings formation of *M. japonicus* and other crustaceans.

## Figures and Tables

**Table 1:** miRNA data output from two miRNA libraries of epidermal tissues.

Sample	MJ1	MJ2	MJ3	MP1	MP2	MP3
Raw reads	11,762,892	11,762,903	10,042,741	11,728,291	10,753,627	14,776,981
N% > 10%	12,242 (0.10%)	11,449 (0.10%)	9628 (0.10%)	11,295 (0.10%)	10,300 (0.10%)	11,720 (0.08%)
Low quality	17,612 (0.15%)	17,041 (0.14%)	14,243 (0.14%)	15,837 (0.14%)	17,496 (0.16%)	30,422 (0.21%)
3 adapter null or insert null	35,803 (0.30%)	365,964 (3.11%)	302,569 (3.01%)	193,872 (1.65%)	166,008 (1.54%)	199,760 (1.35%)
5 adapter contamine	31,254 (0.27%)	15,861 (0.13%)	26,057 (0.26%)	35,134 (0.30%)	28,834 (0.27%)	58,353 (0.39%)
ployA/T/G/C	6538 (0.06%)	9144 (0.08%)	53,716 (0.53%)	4204 (0.04%)	9174 (0.09%)	9638 (0.07%)
Clean reads	11,659,443 (99.12%)	11,343,444 (96.43%)	9,636,528 (95.96%)	11,467,949 (97.78%)	10,521,815 (97.84%)	14,467,088 (97.90%)
Match	9,271,883 (78.82%)	9,674,366 (82.24%)	8,822,865 (87.85%)	10,600,620 (90.39%)	9,254,238 (86.06%)	13,167,375 (89.11%)
Q20	99.63%	99.61%	99.60%	99.64%	99.57%	99.51%
Q30	98.64%	98.52%	98.54%	98.68%	98.38%	98.21%

## Data Availability

The data included in this study can be provided on request from the corresponding author.

## Supplementary Materials

The following supporting information can be downloaded at https://www.mdpi.com/article/10.3390/ani15050727/s1, Table S1: Sequences of the primers used in this study. Table S2: Target genes prediction.
